# Abdominal pressure pain thresholds correlate with postoperative pain intensity

**DOI:** 10.1177/00368504251387759

**Published:** 2025-10-16

**Authors:** Erfan Ghanad, Christel Weiss, Niki Taebi, Jasmin Klick, Sophie Staff, Alida Finze, Martin Dusch, Christoph Reissfelder, Martin Schmelz, Cui Yang

**Affiliations:** 1 Department of Surgery, 99045 University Medical Centre Mannheim, Medical Faculty Mannheim, University of Heidelberg, Mannheim, Germany; 2 Department of Anesthesiology, Intensive Care and Pain Medicine, University Medical Centre Mannheim, University of Heidelberg, Heidelberg, Germany; 3 Department of Medical Statistics and Biomathematics, 99045 Medical Faculty Mannheim, Heidelberg University, Mannheim, Germany; 4 Department of Experimental Pain Research, Mannheim Center for Translational Neuroscience (MCTN), Faculty of Medicine Mannheim, 99045 University of Heidelberg, Mannheim, Germany

**Keywords:** Algometry, pain measurements, pain threshold, postoperative pain, abdominal pain

## Abstract

**Objective:**

We aim to investigate the correlation between ongoing pain levels and pain threshold measured by algometry after bariatric surgery.

**Methods:**

A retrospective analysis was conducted on data from 150 patients who underwent bariatric surgery, including 120 who received Roux-en-Y gastric bypass (RYGB) and 30 who underwent sleeve gastrectomy. Shortly after surgery, pain was assessed by the visual analogue scale (VAS) and abdominal algometry. Algometry was performed on the most sensitive abdominal pressure points.

**Results:**

Patients reported moderate pain after surgery, with a mean of VAS score of 5.7. The mean abdominal pain threshold was 9.8N. VAS pain scores correlated with algometry (Spearman correlation coefficient −0.35; *p* < 0.0001). No sex-specific differences were observed in postoperative pain (*p* = 0.45) or algometry (*p* = 0.99). Furthermore, RYGB and sleeve gastrectomy groups did not differ significantly in the Spearman correlation coefficients (*p* = 0.214).

**Conclusions:**

The mild correlation between reduced mechanical pain thresholds and higher levels of ongoing pain confirms the clinical value of sensory pain testing in the postoperative setting and suggests that local mechanical sensitization contributes to ongoing pain.

## Introduction

The assessment of ongoing pain intensity, commonly performed using tools such as the numeric rating scale (NRS), visual analog scale (VAS), or verbal descriptor scale, plays a pivotal role in the management of both acute and chronic pain. These tools are widely used for pain evaluation in both clinical and research settings^1,2^ and regarded as the gold standard for pain assessment.^3^ Quantitative sensory testing offers the main advantage, that stimulus intensities are well controlled,^4^ however, in neuropathic pain sensory phenotyping by quantitative sensory testing does not correlate to ongoing pain.^5,6^ To reflect the multimodal nature of pain, a variety of standardized stimulation-based assessment methods^7,8^—such as pressure algometry and less common thermal, chemical or electrical stimulus—have been developed.^9,10^

Pressure algometry uses well-controlled tonic mechanical stimuli and can be used to determine pain thresholds and supra-threshold pain responses.^11^ In both clinical practice and research, algometry has proven valuable not only for assessing pain but also for evaluating treatment effectiveness.^12^ Studies have shown moderate^2,13^ to strong correlations^14^ between levels of self-reported ongoing pain (e.g. NRS, VAS) and algometric evaluations. Across a range of orthopedic conditions, including lower back pain,^15–17^ neck pain,^18–20^ chronic pelvic pain,^21^ and patellar tendinopathy,^22^ algometry has demonstrated reliability. It has also proven valuable in the assessment of non-orthopedic disorders like fibromyalgia,^9,23,24^ dysmenorrhea,^25,26^ and endometriosis.^27,28^

From a mechanistic perspective, mechanically induced pain is typically heightened when injured tissues are directly assessed, primarily reflecting the degree of peripheral sensitization. In contrast, when non-involved tissues—such as the thenar eminence—are evaluated, the evoked tonic pain response is more indicative of central pain sensitization.

### Mechanical pain sensitivity of healthy tissue: central pain sensitivity

Notably, algometry in healthy tissue has been shown to predict postoperative outcomes. For instance, Tan et al. demonstrated that low preoperative pain pressure thresholds (PPT) on the thenar and heightened anxiety levels are associated with increased postoperative pain intensity and analgesic consumption.^29^ Similarly, reduced PPT in patients undergoing thoracoscopic surgery and total knee arthroplasty have been linked to more severe postoperative pain and higher perioperative opioid requirements.^30,31^

### Mechanical pain sensitivity of injured or inflamed tissue: peripheral pain sensitivity

On the other hand, direct mechanical testing of injured tissue also correlates with pain experience. A retrospective study of 421 children suspected of appendicitis demonstrated that pressure algometry's diagnostic accuracy exceeded many clinical algorithms and was comparable to ultrasound findings in diagnosing appendicitis.^32^ A similar correlation has been reported for both Achilles tendinitis^33,34^ and knee osteoarthritis.^35^

Based on these observations, one would expect that abdominal pain thresholds after abdominal surgery would clearly indicate local mechanical sensitization that would also contribute to ongoing pain. However, the relationship between self-reported pain scales and algometric evaluation in postoperative abdominal surgery remains underexplored. To our knowledge, no previous studies have specifically investigated the association between PPTs and postoperative pain intensity following abdominal surgery.

In our previously published data, we demonstrated that the postoperative abdominal pressure pain threshold after bariatric surgery is approximately 15.7 N ± 7.0 N.^36^ In contrast, healthy participants showed a significantly higher threshold, with values around 33.9 N ± 13.9 N based on our unpublished data, suggesting that these thresholds are sensitized postoperatively.

We hypothesized that postoperative pain intensity, as measured by the VAS, would inversely correlate with pressure pain thresholds assessed through pressure algometry. Mechanistically, such a correlation could be based on peripheral sensitization, but might also indicate central sensitization in the postoperative period.

## Methods

For this retrospective analysis, all patients who underwent bariatric surgery and recruited as part of the APRAS^36–38^ (DRKS00025579) and RAPRAB (DRKS00029078)^38^ trials (between April 2021 and June 2024) were included. The study was approved by the Ethics Committee II of University of Heidelberg on 2 October 2024 (approval number: 2024-878). A data transfer agreement permitted the inclusion of patients from these trials, and the requirement for study-specific written informed consent was waived by the local ethics committee. The study was conducted in accordance with the Helsinki Declaration of 1975, as revised in 2024. The trial was registered in the German Trial Register DRKS (DRKS00032899) on 8 October 2024. Reporting of the study followed the guidelines outlined in the RECORD statement.^39^ This study was performed at the University Hospital Mannheim.

We assessed abdominal pressure pain thresholds and ongoing pain levels in patients undergoing one of the two most frequently performed surgical metabolic procedures: laparoscopic Roux-en-Y gastric bypass (RYGB) and laparoscopic sleeve gastrectomy (SG).^40^ SG involves longitudinal resection of the greater curvature of the stomach, creating a tubular gastric remnant. RYGB, by contrast, is a more complex procedure that combines the creation of a small gastric pouch with rerouting of the small intestine. Due to its greater degree of visceral manipulation and longer operative times, RYGB is generally considered more invasive than SG.

Each participant was assigned to a unique, randomly generated alphanumeric code that was used throughout data analysis and reporting. A separate, securely encrypted file enabling re-pseudonymisation was stored on a password-protected institutional server. Pain evaluation was conducted before any analgesic intervention. Patients were assessed on the day of surgery on the ward. Pain levels were measured using the VAS.^41^ According to the APRAS^36–38^ and RAPRAB^38^ trial protocols, only patients with a postoperative VAS score ≥3 (0 = no pain, 10 = maximum pain) on the day of surgery while on the ward were eligible. Patients were required to have sufficient communication skills and were excluded if they suffered from chronic pain or had relevant psychiatric disorders. All patients who participated in the above-mentioned trials and underwent bariatric surgery were included. For algometric assessment, the most pressure-sensitive abdominal point was identified and measured.^36,37^ Pain thresholds were determined using an electronic algometer (PCE Instruments FM-200), with results expressed in Newtons (N). The algometer's compression gauge was calibrated by the manufacturer. Participants were positioned supine during the assessments, and force was applied at a constant rate. Each measurement was repeated three times. Details of algometric pain assessment methods have been described in our previous publications^36,37^ and by Tüfekçi et al. for pelvic pain algometry.^42^ The assessments were conducted by four trained raters, who underwent training in algometry prior to performing their first measurements and followed a uniform measurement protocol to ensure consistency across assessments and to mitigate rater variability.

### Statistical analysis

The methods of data collection were carried out in accordance with the guidelines for Good Clinical Practice. Pseudonymized data were entered into an internal REDCap (Research Electronic Data Capture) database by trained research team members. Statistical analysis was created using the SAS software (version 9.4; SAS Institute, Cary, North Carolina, USA). Figures were performed by using GraphPad Prism 10.3. Quantitative variables are presented as mean ± standard deviation. For qualitative factors, absolute and relative frequencies are given. In relevant cases, an unpaired 2-sample t-test was employed to compare the mean values of the two subgroups. For ordinally scaled data, the Mann–Whitney U test (Wilcoxon rank-sum test) was used to compare two independent groups. To compare the two subgroups regarding a categorical variable, a Chi-square test was conducted. Additionally, Spearman's correlation coefficients were calculated to evaluate the strength of the relationship between algometry and VAS. The correlation coefficients of the two subgroups were then compared using Fisher's z-transformation.^43^ To compare the algometry values across the three repeated measurements, a paired Friedman test was applied as a non-parametric test for repeated measures. The result of a statistical test was considered statistically significant if the *p*-value was less than 0.05.

## Results

Table 1 presents the basic characteristics of the 150 patients included in this retrospective analysis, showing no significant differences in age (*p* = 0.6) or BMI (*p* = 0.35) between sexes. Two different types of bariatric surgeries were performed: RYGB and sleeve gastrectomies.

**Table 1.** Basic characteristics.

	Female	Male	Total
Sample size, *n* (%)	120 (80)	30 (20)	150
Age (years ± SD)	43.1 ± 11.6	43.6 ± 10.6	43.2 ± 11.3
BMI (kg/m^2^ ± SD)	45.4 ± 6.1	45.8 ± 6.9	45.5 ± 6.3
Bariatric surgeries (counts):			
—RYGB: Sample size, *n* (%)	100 (85)	17 (15)	117 (78)
—SG: Sample size, *n* (%)	20 (69)	13 (31)	33 (22)

SD: standard deviation; BMI: body mass index; RYGB: Roux-en-Y gastric bypass; SG: sleeve gastrectomy.

### Visual analogue scale

The mean VAS score, assessed immediately before the algometric measurement post-surgery, was 5.7 (range: 3–10). No significant sex effect in the pain score was observed (Wilcoxon two-sample test; *p* = 0.49).

### Algometry

The first algometry measurement consistently showed the highest pain threshold among the three repetitions, although no significant differences were found between the three measurements (measurement 1 vs 2: *p* = 0.2075, measurement 1 vs 3: *p* = 0.5280, measurement 2 vs 3: *p* = 0.9621; Friedman test: *p* = 0.52). Moreover, no sex-specific differences were observed in the algometric measurements (*p* = 0.99). The mean algometric measurement was 9.8 N (compare Table 2).

**Table 2.** Correlation between algometry and VAS in bariatric patients.

	Mean [*N*]	Min/Max [*N*]	Std Dev [*N*]	Spearman coefficient	*p*-value^a^
1. Measurement	11.0	1.2/41.3	7.3	−0.308	0.0001
2. Measurement	9.7	1.4/28.6	6.0	−0.323	<0.0002
3. Measurement	9.7	1.4/31.6	6.1	−0.338	<0.0001
Average	9.8	1.4/30.1	6	−0.348	<0.0001

Mean VAS score, recorded immediately prior to performing algometry, was 5.7 and was used for the correlation analysis.

N: Newton.

a *p*-value of the Spearman correlation coefficient tests for the correlation VAS score and algometry.

### Correlation between VAS and pressure algometry

**Figure 1.**
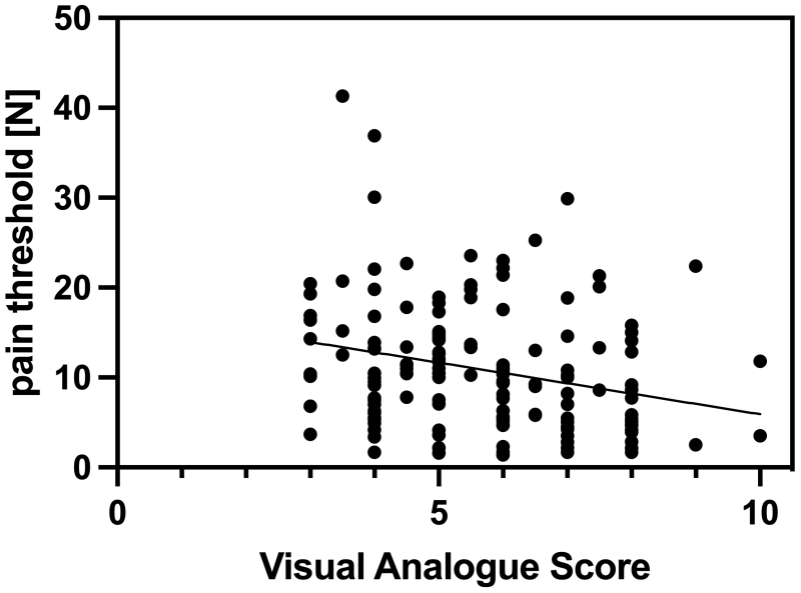
Correlation visual analogue score and pain threshold (N) with included regression line (*r* = −0.35, *p*-value <0.0001).

The correlation between self-reported pain scores and algometry was analyzed using Spearman correlation coefficients. A significant inverse relationship was found between levels of ongoing pain (VAS scores) and mechanical pain thresholds (algometry): The Spearman correlation coefficient of −0.35 showed a moderate correlation (*p* < 0.0001), indicating that higher pain scores were associated with lower pressure threshold (Figure 1, Table 2). There was no significant association between pain score and sex (*p* = 0.4908), age (*p* = 0.6043), or type of surgery (*p* = 0.33).

#### Subgroup analysis

The median pain scores for RYGB and SG were 5.7 and 6.0, respectively. The Mann–Whitney U test showed no significant difference in pain scores between the two surgical types (*p* = 0.33). Similarly, algoemetric assessments revealed no significant difference between the groups (*p* = 0.08).

Spearman correlation analysis demonstrated a significant moderate inverse correlation between VAS scores and algometry measurements, with a correlation coefficient of −0.30092 (*p* = 0.0016) for RYGB and −0.53845 (*p* = 0.0055) for SG, indicating that higher pain scores were associated with lower pressure pain thresholds (see Tables 3 and 4). The difference between the correlation coefficients was not statistically significant (*p* = 0.21389).

**Table 3.** Correlation between VAS and algometry for patients undergoing RYGB.

	Mean [*N*]	Min/Max [*N*]	Std Dev [*N*]	Spearman coefficient	*p*-value^a^
1. Measurement	10.4	1.2/36.9	6.9	−0.311	0.0006
2. Measurement	9.5	1.4/28.6	5.9	−0.284	0.0029
3. Measurement	9.5	1.4/31.6	6	−0.296	0.002
Average	9.6	1.4/30.1	6	−0301	0.002

Mean VAS score, recorded immediately prior to performing algometry, was 5.7 and was used for the correlation analysis.

N: Newton.

a *p*-value of the Spearman correlation coefficient tests for the correlation VAS score and algometry.

**Table 4.** Correlation between VAS and algometry for patients undergoing sleeve gastrectomy.

	Mean [*N*]	Min/Max [*N*]	Std Dev [*N*]	Spearman coefficient	*p*-value^a^
1. Measurement	13.2	2.6/41.3	8.4	−0.356	0.04
2. Measurement	10.5	3.0/22.6	6.2	−0.49	0.01
3. Measurement	10.6	3.0/22.3	6.5	−0.512	0.008
Average	10.8	2.9/23.0	6.5	−0.538	0.006

Mean VAS score, recorded immediately prior to performing algometry, was 6.0 and was used for the correlation analysis.

N: Newton.

a *p*-value of the Spearman correlation coefficient tests for the correlation VAS score and algometry.

## Discussion

In this study, we investigated the correlation between a self-reported unidimensional pain score, such as the VAS, and pain provocation tests using algometry, identifying a mild but significant inverse correlation between VAS scores and algometric assessment. To the best of our knowledge, this is the first evaluation of correlation between VAS and pressure algometry in the postoperative setting after abdominal surgery. Abdominal surgeries often involve both visceral and somatic pain components, leading to more complex pain experiences.

Integrating objective assessment methods, like algometry, into a multidimensional approach can enhance pain understanding, improve management strategies, and support advancements in both clinical practice and research.^2^ Preoperative pain threshold assessments have been shown to correlate with both postoperative pain intensity and analgesic consumption.^44^ In a study involving patients undergoing lung cancer surgery, the perioperative pain thresholds were found to predict postoperative pain levels.^45^ These findings suggest that pain threshold assessment may serve as a valuable tool for identifying patients at risk of developing severe postsurgical pain.^45,46^

Our findings are consistent with previous studies. A negative correlation between pain and pain threshold has been reported by Wang et al., who demonstrated a similar relationship between pain threshold and intensity in patients experiencing acute pain after thoracic surgery.^30^ In contrast, Sanches et al. found no correlation between pressure pain thresholds and pain intensity in patients with temporomandibular disorder.^12^ Notably, their study utilized standardized trigger point locations without assessing the most sensitive areas. With our study, we were able to demonstrate that the sensitivity of trigger points varies based on the type of surgery: bariatric surgery patients exhibited increased sensitivity in the upper abdomen, while intestinal surgery patients showed greater sensitivity in the lower abdomen.^37^ This suggests that assessing the most sensitive trigger points prior to algometry may be crucial for accurately identifying the locus dolendi. Additionally, the difference in patient populations may explain the contrasting results. Sanches et al.'s study included patients with chronic pain, which involves different mechanisms compared to the acute postoperative pain observed in our study.^47^ Postoperative pain involves the activation of nociceptors by inflammatory mediators which might lower the pain thresholds and increase neuronal excitability, ultimately leading to hyperalgesia or even chronic pain.^48,49^

In the study conducted by Chesterton et al., a sex disparity was observed in the assessment of pressure pain thresholds at the first interosseous muscle.^50^ However, our study did not show any sex-specific differences in pain scores measured via VAS or in algometric assessments. This may be due to the significantly higher proportion of women (74.3%) compared to men (25.7%) included in our study. Previous studies show that differences in descending pain inhibition can predispose women to enhanced central excitability in both acute and chronic settings.^51,52^ Such mechanisms could conceivably be triggered in the early postoperative period, but were not specifically assessed here. Future work will be necessary to clarify whether sex-dependent central sensitization contributes to postoperative pain variability.

Although the first algometric measurement in our study was slightly elevated, no significant differences were observed compared to subsequent repeated values. Therefore, excluding the first value or using only one value, as done in other studies,^53,54^ did not appear necessary.

Our study has several limitations. First, different raters were involved, each assessing varying numbers of patients, and since each patient was evaluated by only one rater, interrater reliability could not be assessed. Although we demonstrated a negative correlation between VAS scores and pain thresholds, the reproducibility of our findings across different raters remains uncertain. However, involving multiple raters immediately post-surgery would have imposed considerable demands on patients and may not have been feasible. Furthermore, a significantly higher proportion of female patients were evaluated, making it more challenging to investigate potential sex-specific differences in pain perception. In addition, this retrospective analysis of local pain threshold assessment may suggest a role for peripheral pain sensitization due to tissue injury or inflammation. However, due to the heterogeneity of the included patient population and the study design, no definitive conclusions can be drawn regarding the underlying pain mechanisms, particularly as central sensitization was not specifically evaluated.^19^

It's vital to remember that at this time, there isn’t a single tool that can accurately and completely assess pain. Combining provocation tests like algometry with self-reported unidimensional tools can offer a more detailed understanding of pain, facilitating better clinical evaluations and enhancing research comparability. This approach may provide an additional semi-objective tool for assessing pain, offering a valuable parameter for therapeutic monitoring within a multimodal assessment framework. Moreover, this tool could be particularly beneficial for patients with language barriers, enhancing accessibility and understanding in pain evaluation. Therapeutically, algometry could provide a valuable tool for assessing postoperative outcomes and predicting pain levels following surgery.^44^ While a low preoperative pain threshold has been associated with increased postoperative pain intensity, no clear correlation with chronic postoperative pain has been established.^55^ In contrast, the potential association between postoperative pain threshold assessments and the development of chronic postoperative pain has not yet been investigated.

## Conclusion

This retrospective analysis demonstrated a mild negative correlation between pain scores measured by the VAS and algometry for acute pain following bariatric surgery. Consequently, these findings suggest that local mechanical sensitization may contribute to postoperative pain. By utilizing algometry, researchers and clinicians can obtain more standardized and reproducible measurements of pain. This approach might minimize the influence of subjective bias and provides an additional tool for comprehensive multimodal pain assessment.
